# Barriers to substance use disorder treatment among Asian Americans in New York City: A qualitative study of provider perspectives

**DOI:** 10.1371/journal.pmen.0000743

**Published:** 2026-10-21

**Authors:** Sueun Hong, Megan Sanico, Haofan Yang, Stella S. Yi, Sahnah Lim, Charles J. Neighbors, Sugy Choi

**Affiliations:** 1 Department of Population Health, New York University Grossman School of Medicine, New York, New York, United States of America; 2 School of Public Health, University of Illinois Chicago, Chicago, Illinois, United States of America; 3 Department of Health Policy and Management, Emory Rollins School of Public Health, Atlanta, Georgia, United States of America; PLOS: Public Library of Science, UNITED KINGDOM OF GREAT BRITAIN AND NORTHERN IRELAND

## Abstract

Substance use disorders (SUDs) are a major mental health and public health concern in the United States, yet research and services for Asian American populations remain sparse. Although SUD prevalence among Asian Americans is not consistently higher than in other racial groups, treatment utilization is markedly lower, and the aggregation of diverse ethnic groups into a single category and the “model minority” stereotype obscure heterogeneity in needs and contribute to underdiagnosis, undertreatment, and limited culturally competent care. We conducted one-on-one, semi-structured interviews with eight healthcare and community-based providers serving Asian American clients with SUD across clinical and community settings in New York City (NYC) between December 2022 and May 2023. Participants were recruited through purposive sampling. Interviews lasted 45–60 minutes, were audio-recorded and transcribed verbatim, and were analyzed using a combined deductive–inductive thematic approach, alongside multiple researchers independently coding the data and reconciling themes through team-based discussion. Six interrelated themes emerged: types of services provided; substance use trends; barriers to care; community needs; existing interventions tailored to Asian Americans; and client engagement strategies. Providers described a wide spectrum of inpatient, outpatient, and court-mandated services, but identified pervasive shortfalls in cultural and linguistic competence. Substance use patterns differed by age, with growing ketamine use among younger clients and persistent alcohol use among older clients. Key barriers included stigma rooted in cultural beliefs about mental health, limited bilingual capacity, opaque communication from court-mandated treatment pathways, and intersecting socioeconomic pressures including housing instability and food insecurity. Providers also noted that aggregation of Asian American subgroups in data collection obscured distinct needs and impeded program planning. Findings highlight a pressing need for culturally and linguistically tailored SUD treatment, stronger collaboration between treatment programs and the legal system, sustained investment in community-based organizations, and disaggregated data collection across Asian American subgroups.

## Introduction

Substance use disorders (SUDs) are among the most consequential mental health conditions in the United States, contributing to substantial morbidity, mortality, and reduced quality of life [1]. The 2023 National Survey on Drug Use and Health estimated that 48.4 million people aged 12 years or older met criteria for an SUD in the previous year [2]. SUDs frequently co-occur with other mental health conditions and are shaped by overlapping social, cultural, and economic determinants [1,3]. Yet within mental health and addiction research, Asian American populations remain markedly understudied, contributing to persistent disparities in treatment access and quality [4–7].

Asian Americans are among the fastest-growing racial and ethnic groups in the United States, with an estimated 22 million residents in 2019 and a projected population of 46 million by 2060 [8]. Despite this growth, prior work shows that Asian Americans are consistently underrepresented in clinical and health services research and that disaggregated data on Asian ethnic subgroups are rarely collected [4,6,9]. Although SUD prevalence among Asian Americans is not, on aggregate, higher than among non-Hispanic White populations, Asian Americans have markedly lower rates of SUD treatment utilization [10]. They are also approximately 50% less likely than other racial groups to seek mental health services more broadly, with cultural stigma and a shortage of culturally responsive care among the most frequently cited deterrents [11].

The “model minority” stereotype—the assumption that Asian Americans uniformly experience favorable health, economic, and social outcomes—masks substantial heterogeneity in needs across subgroups and contributes to underdiagnosis and undertreatment of mental health and substance use conditions [9,12,13]. Asian American adults comprise dozens of ethnic groups with distinct cultural, linguistic, and historical contexts, and prevalence of SUD varies meaningfully across subgroups [14–16]. Cultural beliefs about mental health, intergenerational expectations, and limited culturally tailored services further deter help-seeking, while Asian Americans also report fewer positive interactions with healthcare providers and are less likely to receive counseling [17]. Together, these dynamics underscore the need for culturally informed strategies that account for within-group diversity and the structural conditions shaping mental health [18,19].

Research on SUD and co-occurring mental health needs in Asian American communities remains sparse [4,5,10], and few studies have centered the perspectives of providers who deliver care to this population [10,14]. A recent systematic review of substance use and treatment disparities among Asian Americans, Native Hawaiians, and Pacific Islanders documented not only the paucity of studies but also the frequent failure to disaggregate ethnic subgroups, obscuring meaningful within-group variation [4]. This gap is compounded at a structural level: analyses of NIH-funded clinical research show that Asian American, Native Hawaiian, and Pacific Islander participants have been persistently underrepresented across nearly three decades of federally funded studies. The ‘mutually reinforcing cycle of poor data quality and racialized stereotypes’—in which the model minority framing and aggregation practices reduce both the perceived need for and the actual conduct of research—further entrenches these evidentiary gaps.

Moreover, few studies have centered the perspectives of the providers who deliver care to this population. Foundational work on Asian Americans, addiction, and barriers to treatment has long argued that clinician-facing insights are essential to understanding engagement and retention, yet provider-level qualitative research remains limited [20]. Broader calls to action in Asian American mental health have emphasized that understanding help-seeking, stigma, and service utilization requires attention to the cultural and systemic contexts in which providers operate, and studies of Asian Americans’ healthcare experiences point to fewer positive provider interactions and lower rates of counseling. Because provider-level insights can illuminate cultural, linguistic, and systemic obstacles to engagement—and can identify leverage points for culturally responsive intervention—the relative absence of such research represents a critical gap that the present study aims to address.

## Materials and methods

### Ethics statement

The study was approved by the NYU Langone Health Institutional Review Board (IRB protocol #S22-00857) and conducted in accordance with the principles of the Declaration of Helsinki. The IRB approved the use of verbal informed consent, with a waiver of documentation (written signature) of consent, given the minimal-risk nature of the interviews and their conduct via remote (Zoom) and in-person modalities. Before each interview, the interviewer (SH or SC) read a standardized, IRB-approved consent script that reviewed the study’s purpose, procedures, potential risks and benefits, confidentiality protections, and the voluntary nature of participation, and answered any questions; participants then provided verbal consent both to take part and to be audio-recorded. Consistent with the IRB-approved waiver of documentation, no signed consent form or separate witness attestation was obtained; instead, the interviewer obtained verbal consent directly and, for each participant, noted within the study records that verbal consent had been provided. Written signatures were not collected in order to limit the retention of identifying information. Participants were assured that their responses would be kept confidential, and no identifying information about participants or their clients is reported.

### Study design

We used a qualitative study design to explore how providers serving Asian American clients with SUD in NYC describe service provision, barriers to care, community needs, and client engagement strategies. A qualitative approach was selected to surface cultural, linguistic, and systemic dimensions of care that are difficult to capture in quantitative research and that have been underexplored in prior work on Asian American mental health and addiction services [21]. The study was guided by the research question: how do service provision, barriers to care, community needs, and client engagement strategies shape access to SUD treatment for Asian American communities in NYC. Reporting follows the Consolidated Criteria for Reporting Qualitative Research (COREQ) checklist (S1 COREQ Checklist).

### Participants and setting

Participants were healthcare and community-based providers currently serving Asian American clients with SUD across clinical and community settings in NYC, including clinic directors, behavioral health clinicians, counselors, a community outreach coordinator, a certified recovery peer advocate, and an intern (n = 8; see Table 1). We used purposive sampling, with recruitment facilitated by the NYU Center for the Study of Asian American Health (CSAAH; NIH U54MD000538), which referred professionals with direct experience treating this population. From these referrals, we identified eligible providers through email screening; we also disseminated study flyers describing the purpose of the research. To be eligible, providers had to be currently working directly with Asian American clients in NYC. Interviews were conducted between December 2022 and May 2023 by two members of the study team (SH, SC). Participating settings included hospital-based programs, outpatient SUD treatment programs, and community-based organizations (CBOs); several offered linguistically appropriate services in Korean and Mandarin.

**Table 1:** Sociodemographic characteristics of interview participants (n = 8).

Baseline characteristic	Asian	Not Asian
	*n*	*n*
**Gender**		
Female	6	1
Male	1	0
**Occupation**		
Director	3	0
Behavioral health clinician	2	0
Community outreach coordinator	1	0
Certified recovery peer advocate	0	1
Intern	1	0
**Total**	**7**	**1**

Cells indicate counts (n) of participants. Numbers may not sum to 8 due to category structure.

### Data collection procedures

We conducted one-on-one, semi-structured interviews that explored four core topics: service provision, barriers to care, community needs, and client engagement strategies. Interview guides were developed by the study team (S1 Text) and adapted iteratively to capture relevant insights from each participant. Interviews lasted approximately 45–60 minutes and were conducted in person or via Zoom according to participant preference. Verbal informed consent was obtained at the start of each interview, and participants were assured that responses would be kept confidential. All interviews were audio-recorded with consent and transcribed verbatim. Personal identifiers were removed during transcription. Consistent with prior work indicating that thematic saturation can be reached with as few as six interviews [22], we observed saturation after six interviews and conducted two additional interviews to confirm that no new themes were emerging.

### Data analysis

We analyzed the interview transcripts using a combined deductive–inductive thematic approach [23]. Broad topic domains corresponding to the four areas covered by the interview guide—service provision, barriers to care, community needs, and client engagement strategies—were specified a priori and provided an initial organizing framework for the analysis. Within and across these domains, specific codes, themes, and subthemes were not predetermined but were developed inductively from the data during coding. Field memos were written after each interview to document early impressions, emerging concepts, and indicators of saturation. Three researchers (SH, MS, HY) independently coded the transcripts to enhance reliability, generating initial participant-level matrices that were reviewed and reconciled during team meetings. Through iterative discussion, we consolidated these matrices into a single matrix that organized the inductively derived themes within the topic domains. Disagreements in coding were resolved by consensus.

## Results

Eight providers participated in semi-structured interviews. Participant demographics and roles are summarized in Table 1. Six analytic themes emerged from the data: (1) types of services; (2) substance use trends; (3) barriers to care; (4) community needs; (5) existing interventions tailored to Asian Americans; and (6) client engagement strategies. A summary of themes and key points is presented in Table 2.

**Table 2:** Themes and key points from interviews with healthcare and community-based providers (n = 8).

Theme	Key points
**Types of services**	• Inpatient and outpatient treatment; screenings; and care for high-risk and court-mandated clients. • Referrals to other medical and mental health services. • Hospital-based programs with dedicated floors or services for Asian American populations.
**Substance use trends**	• Rising ketamine use among younger Asian American clients. • Persistent alcohol use among older clients. • Many clients do not perceive their use as indicative of a substance use disorder. • Socioeconomic stressors (e.g., demanding work schedules, financial insecurity) shape use and complicate engagement.
**Barriers to care**	• Cultural stigma and differing belief systems lead to underutilization; clients fear judgment from family or community. • Preference for providers who share clients’ language or background, alongside a shortage of bilingual providers and interpreters. • Limited higher-level care available for non–English-speaking Asian clients. • Unclear court expectations and communication complicate court-mandated treatment. • Housing instability, food insecurity, and economic precarity divert focus from recovery.
**Community needs**	• Limited culturally responsive social support networks; social isolation among first-generation immigrants. • Race/ethnicity data not routinely collected, and used more for language matching or program assignment than for monitoring outcomes. • Aggregation of Asian American subgroups obscures distinct community needs.
**Existing interventions tailored to Asian Americans**	• Bilingual services (e.g., Mandarin, Korean, Bengali) enable engagement without interpreters. • CBOs provide culturally tailored outreach, education, and referrals, though constrained by funding cycles and limited resources. • Court-mandated programs are a common pathway into treatment; stronger legal–treatment collaboration is needed.
**Client engagement strategies**	• Trust is built over time; clients often do not disclose use immediately. • Community outreach used to engage clients before crises develop. • Early intervention and consistent, culturally informed outreach help reduce premature termination.

### Types of services

Providers described a broad portfolio of services delivered to Asian American clients with SUD in NYC, including inpatient and outpatient treatment, evaluations for high-risk clients, court-mandated treatment, referrals to other medical and mental health services, and hospital-based programs with dedicated floors or services for Asian American populations. As one director explained:

*“we have a responsibility to make sure that the community members that we serve have access to quality healthcare.”* — Director.

Participants emphasized that culturally and linguistically appropriate care—delivered by providers who recognized cultural nuance—enhanced client trust and engagement. The breadth of services available reflects ongoing efforts to expand access and retention for clients facing multiple systemic obstacles.

### Substance use trends

Providers identified shifting patterns of substance use across age cohorts. Ketamine use was described as increasingly common among younger Asian American clients, while alcohol use remained the dominant concern among older clients—a pattern consistent with prior ethnographic work on Asian American substance use [24]. A behavioral health clinician noted:

*“I was so astonished when I’m going through charts, and I’m looking to see how many of the clients are addicted to ketamine, and it is alarming. … A lot of them are alcoholics or use other drugs, but this ketamine thing got them.”* — Behavioral Health Clinician.

Participants also emphasized the role of socioeconomic stressors in shaping substance use. Demanding work schedules, financial insecurity, and limited time for treatment engagement complicated recovery efforts. As one provider observed:

*“I feel a lot of times when I work with Asian, immigrant clients they do have a lot of pressure to make ends meet first, and they have very tight work schedules.”* — Behavioral Health Clinician.

Providers suggested that rising ketamine use among younger clients may reflect its accessibility and popularity in nightlife settings, while continued alcohol use among older clients may be reinforced by cultural acceptance of alcohol as a social lubricant.

### Barriers to care

Providers identified multiple, often interrelated barriers to SUD care: cultural stigma around mental health and substance use, language and communication challenges, legal system entanglements, and economic precarity. As one counselor summarized:

*“They couldn’t even find shelter and food, how would they be able to attend to their mental health needs? … I just don’t think it’s possible to dig too much deeper into mental health if this person’s worried about what to eat tomorrow.”* — Counselor, SUD clinic.

#### Cultural stigma.

Stigma rooted in cultural beliefs about mental health and substance use was described as a pervasive barrier. Many clients feared judgment from family or community members and delayed or avoided treatment as a result; in some cases, normalization of behaviors such as binge drinking or nicotine use further discouraged help-seeking. As one director described:

*“I think sometimes, because of the patriarchal nature of some of our cultures, we don’t delve too deeply into some of the healthcare challenges or some of the SUD challenges.”* — Director.

#### Language and communication.

Language and communication issues were a second major barrier, particularly among first-generation immigrants. Some clients struggled to navigate the healthcare system due to limited English proficiency and preferred to receive care in their native languages. However, a shortage of bilingual providers and interpreters limited access to linguistically concordant care. A community outreach coordinator observed:

*“They’re already in the hospital, they feel unsafe, uncomfortable, but if the people speak the same language, it helps them express what’s happened, and then we are able to understand cultural dynamics.”* — Community Outreach Coordinator.

#### Legal involvement, housing, and economic precarity.

Legal system involvement, housing instability, and economic precarity emerged as deeply intertwined structural barriers. Many clients were court-mandated to attend treatment, and providers reported inconsistent communication from the legal system that left both clients and clinicians struggling to navigate compliance. A counselor at an SUD clinic noted:

*“I wish the court could give more clear direction and instruction to the client and to us … And I wish there was a better way to communicate to translate to help clients to help understand the situation and explain to them how the system works.”* — Counselor, SUD clinic.

Housing instability further complicated sustained treatment engagement. A director described how broader social and economic conditions in NYC strained the ability of clients to focus on recovery:

*“I think we are seeing a lot of social and economic challenges, obviously. And I think we continue to see so much homelessness. We continue to see folks that are outpriced in terms of what’s affordable. … In New York State, our minimum wage is $15.00 an hour but that really is not a living wage in New York City.”* — Director.

Economic hardship and food insecurity were described as worsening since the COVID-19 pandemic, despite ongoing advocacy. The same director noted:

*“People not being able to afford food. And through the pandemic, we are seeing slowly through surveying and whatnot that food insecurity and providing basic needs is increasing. I don’t see it getting better. I see it getting worse despite all the advocacy.”* — Director.

### Community needs

Providers described a notable absence of culturally responsive social support networks. First-generation immigrants in particular reported social isolation, intra-family communication difficulties, and limited access to resources attuned to their cultural and socioeconomic circumstances. One clinician reflected:

*“I feel for the clients I serve, they do not have this type of community support. Maybe they work in a restaurant they’re surrounded by other Chinese folks, but maybe they do not feel so integrated into the whole society. Maybe their children speak English at home and they do not feel they can communicate with their children.”* — Behavioral Health Clinician.

Providers also emphasized that distinct Asian American subgroups have distinct needs and that aggregating them into a single category obscured these differences. As one director put it, “All community members are coming in for services. It’s going to be [important] to understand so that we’re not repeating the work and that we have a better grasp of where our community’s coming from and what the needs are.” Across interviews, participants framed social determinants—economic stability, social connection, and mental health support—as central to improving treatment access and retention.

### Existing interventions tailored to Asian Americans

Several programs were identified as offering culturally and linguistically tailored services. Bilingual services in Mandarin, Korean, and Bengali allowed clients to engage in treatment without relying on interpreters. As one community outreach coordinator described:

*“So the social worker will be the one in charge of everything … But if it’s Chinese they were specific to that social worker. We also have a Korean speaking so we have a Korean social worker here who takes specific language.”* — Community Outreach Coordinator.

CBOs in some neighborhoods provided outreach, education, and referrals, although funding limitations constrained their reach. Court-mandated programs were among the most common pathways into treatment for Asian American clients; while these offered structured support, providers stressed the need for stronger collaboration between the legal system and treatment centers to improve compliance and outcomes. As one counselor reflected:

*“So the court also wants us to give them a treatment recommendation after seeing clients for two assessments. … How can a client who doesn’t really understand what therapy is set a measurable treatment goal?”* — Counselor, SUD clinic.

### Client engagement strategies

Engaging Asian American clients in SUD treatment remained a substantial challenge. Stigma, fear of legal consequences, and reluctance to discuss substance use contributed to delayed presentation and high attrition. Providers emphasized the importance of early intervention and consistent, culturally informed outreach. As one clinician described:

*“Even if they walk in our door, it doesn’t mean they’re ready for treatment. … So I feel it’s really how to engage clients to come to the service and help them decide to stay in the service. The engagement part is huge because without them coming here regularly, change cannot happen. And we do see the drop-out rate is pretty high, or the premature termination is also high compared with maybe other types of service or other populations.”* — Behavioral Health Clinician.

Participants viewed culturally competent community outreach as a key strategy for reducing premature termination and supporting sustained engagement.

## Discussion

This qualitative study of eight providers serving Asian American clients with SUD in NYC identifies a pressing need for culturally informed and accessible SUD treatment for this underserved population. Across diverse settings, providers described a healthcare landscape that offered a broad range of services—from inpatient and outpatient programs to court-mandated treatment—yet exhibited substantial gaps in cultural and linguistic adaptation. The strong preference among clients for linguistically concordant and culturally aligned providers, and the barriers reported by non-English speaking Asian clients, underscore the centrality of cultural competence in SUD and mental health care. These observations align with prior evidence that culturally responsive care builds trust and engagement, particularly in communities with significant stigma around mental health and substance use [25,26].

A recurring theme was the underrecognition of SUD by clients themselves, often shaped by cultural norms that normalize certain substance use behaviors [14]. Combined with stigma around mental health, this contributed to low disclosure and underutilization of services. Building therapeutic trust required substantial time and was complicated when providers were unfamiliar with the cultural contexts of their clients. Rising concerns about housing instability and economic insecurity within Asian American communities further compounded these barriers, as clients prioritized immediate survival needs over engaging in mental health or SUD treatment [27]. Providers also expressed frustration about communication gaps with the legal system in the context of court-mandated treatment, echoing prior work on the importance of integrated, high-touch management for complex cases [28].

The challenges providers described mirror broader trends in the social determinants of mental health, in which structural forces—employment conditions, immigration policy, housing affordability, and racism—shape who can access and benefit from care [19]. Financial constraints on CBOs were a particularly salient concern. Budget limitations restricted recruitment of qualified bilingual staff, implementation of culturally tailored treatment protocols, and maintenance of community engagement programs, contributing to persistent inequities in access [29].

Variability in subgroup data collection revealed both strengths and gaps in current practice. Some programs had defined reporting processes; others did not. Race and ethnicity data, when collected, were often used for language matching or program assignment rather than for systematic monitoring of disparities [1]. The aggregation of diverse Asian subgroups into a single racial category masks meaningful differences in health behaviors, mental health needs, and treatment outcomes, contributing to insufficiently targeted interventions [9]. Disaggregated data collection and outcome tracking across demographic variables are necessary to bridge these gaps and inform culturally responsive service design.

Healthcare disparities research consistently demonstrates that mainstream treatment models often privilege dominant racial groups, leaving minority subgroups with limited tailored options. For Asian American communities, expanded financial support for CBOs, technical assistance for culturally responsive program development, and stronger collaboration across the legal system, treatment programs, and community organizations represent priority areas for change. Addressing these challenges will require a comprehensive strategy that links early intervention with sustained resource allocation to overcome obstacles related to legal involvement, housing instability, and economic precarity.

### Limitations

This study has several limitations. First, although we observed thematic saturation after six interviews and conducted two additional interviews to confirm it, the sample of eight providers is small and recruited from a single metropolitan area, limiting transferability. Regional differences in Asian American population composition, available services, and state policies likely produce distinct treatment environments, and similar work in other metropolitan settings is warranted. Second, the study did not directly examine client experiences; client voices remain a critical complement to provider perspectives and should be incorporated in future work. Third, we did not fully address intersections of race with gender, sexual orientation, immigration status, and socioeconomic status. Specific challenges faced by women, LGBTQ+ individuals, and recent immigrants within Asian American communities warrant dedicated investigation. Fourth, longitudinal data are needed to characterize how culturally tailored interventions affect recovery trajectories and downstream mental health outcomes. Finally, while this study highlights provider-perceived obstacles, additional research is needed to understand how external constraints—funding structures, insurance design, and policy environments—shape service quality and accessibility.

## Conclusion

Provider perspectives reveal that improving SUD treatment for Asian Americans in NYC requires nuanced, culturally informed approaches embedded within a stronger mental health and social services infrastructure. Despite a substantial range of available services, providers identified persistent gaps in cultural and linguistic competence, limited disaggregated data on Asian American subgroups, and an insufficient infrastructure of culturally tailored programs. Cultural considerations—particularly mental health stigma, varying acculturation levels, and the importance of trust—emerged as central to engagement and retention.

Patterns of substance use, including rising ketamine use among younger clients and continued alcohol use among older clients, point to the need for age- and subgroup-specific outreach and early intervention. Although community outreach programs targeting high-risk populations are a promising direction, systemic challenges—including constrained CBO funding, fragmented coordination with the legal system, and intersecting socioeconomic pressures—remain unresolved. Future work should integrate client perspectives, examine intersectional needs, expand disaggregated data collection, and evaluate culturally tailored interventions in diverse regions to advance equitable mental health and SUD care for Asian American communities.

## Supporting information

S1 COREQ Checklist Consolidated Criteria for Reporting Qualitative Research (COREQ) 32-item checklist. (PDF)

S1 Text Semi-structured interview guide used with healthcare and community-based providers. (DOCX)
