# Supplementary material for: Barriers to substance use disorder treatment among Asian Americans in New York City: A qualitative study of provider perspectives
Source: PLOS Ment Health. 2026 Oct 21;3(10):e0000743. doi: 10.1371/journal.pmen.0000743 (PMC13686486; doi:10.1371/journal.pmen.0000743)
Supplement: Supplementary file 2 [file pmen.0000743.s002.docx]

[*Note: As explained in the protocol, this is a semi-structured guide, questions may arise that are not included, but will be directly related to the topics and themes included here.]*

**Interviewer ID** _________

1. **Interview Date (DD/MM/YYYY)**	**__________________**
2. **Time Start**	**_______**

**Thank you for agreeing to participate in our interview. As we mentioned before, we are exploring ways to improve quality of Asian American and subgroup data by interviewing key informants who work with Asian populations. We are very interested in learning about your experience here at (name of clinic).**

1. **To start, we would like to know a bit about your role as__[title here]__ here at __[name of clinic]__.**
a. **How long have you been with __[name of clinic]__?**

**____________________**

b. **How long have you been in this role at this clinic?**

**____________________**

c. **Have you held a similar role at a different clinic?**

**____________________**

2. **[For SUD treatment clinics ONLY] We’re interested in understanding the type of services your clinic provides and how it operates today. Describe what type of services are offered at the [OTP/SUD outpatient] clinic.**

**Probe for:**

a. **Individual and group counseling**
   i. **Therapeutic approaches: (brief intervention, motivational interviewing, trauma-related counseling, cognitive behavior therapy…etc.)**
b. **Delivery of treatment: telehealth/telemedicine**
c. **Medication for opioid use disorder (methadone, buprenorphine, naltrexone…)**
d. **Outpatient (ambulatory) detoxification**
e. **Intensive outpatient treatment**
f. **General medical care (i.e. screenings, annual exams, etc)**

3. **To get a sense of your day-to-day work, walk me through what you do on a regular basis.**

**Probe(s):**

a. **What activities are you engaged in?**
b. **With whom are you working closely?**
   i. **How are you collaborating with these staff members?**
   ii. **Do you meet regularly?**

***Note to interviewer: For question 4*, *if they give a range, either enter the middle of that range or try to get them to give a concrete number.***

***We will now be asking about statistics at the clinic.***

4. **Individuals seeking substance use disorder treatment can vary by age, gender, or other characteristics. Which categories of individuals listed below are served by this facility?**
   a. **Roughly what percentage of the patients that you see have Medicaid? _____%**
   b. **Male? _____ %**
   c. **Asian? _____ %**
      i. **South Asian _____ %**
      ii. **Chinese _____ %**
      iii. **Filipinx _____ %**
      iv. **Vietnamese _____ %**
      v. **Korean _____ %**
      vi. **multi-racial _____ %**
      vii. **other _____ %**
   d. **Black or African American? _____%**
   e. **Hispanic? _____%**
   f. **Other racial-ethnic groups (excluding White)? _____%**
      i. **If so, specify.**
   g. **About what percentage of the clients within your clinic are mandated? _____%**
5. **How comprehensive do you feel that race/ethnicity data is being collected within your clinic or organization?**
   a. **Do you feel that all race/ethnicity categories are well covered?**
   b. **What categories are missing?**
6. **What are the main reasons/goals behind collecting race/ethnicity data at your clinic?**
7. **Who are the individuals who decided to collect race/ethnicity data?**
8. **What is the process for collecting race/ethnicity data for patients at your clinic?**

**Probe for:**

a. **When do you collect race/ethnicity data?**
b. **Do you collect Asian American subgroup data?**
c. **Do you have any prior experience with collecting race/ethnicity data?**
   i. **Asian American subgroup data?**
d. **How crucial was or is race/ethnicity data collection to the clinic?**
   i. **Asian American subgroup data?**
e. **What kind of value does it add?**
   i. **Asian American subgroup data?**

9. **Does your clinic collect language/heritage data in addition to race/ethnicity?**
   a. **If so, how does your clinic use this information?**
10. **[ONLY if the clinic is collecting Asian American subgroup data] What do you think is the most important part of collecting Asian American subgroup data?**
11. **[If the clinic is NOT collecting Asian American subgroup data] What are your thoughts about collecting Asian American subgroup data?**
    a. **Are there any challenges that this clinic faces with collecting Asian American subgroup data?**
    b. **Do you see any certain strategies that work to mitigate these challenges?**
12. **What kind of audits, checks and monitoring criteria are there to ensure race/ethnicity data collection is going smoothly?**
    a. **Which department deals with all of that?**
13. **Can you describe your clinic’s efforts regarding data quality improvement?**
    a. **Do you or your organization examine outcomes according to different client demographics, for example, by race/ethnicity or gender? If yes, how have you used this data/information?**
14. **Are there diversity, equity, and inclusion initiatives happening within your clinic or organization? If yes, please describe.**
15. **Is there anything that we’ve missed that you’d like to add?**
