# Supplementary material for: Barriers to substance use disorder treatment among Asian Americans in New York City: A qualitative study of provider perspectives
Source: PLOS Ment Health. 2026 Oct 21;3(10):e0000743. doi: 10.1371/journal.pmen.0000743 (PMC13686486; doi:10.1371/journal.pmen.0000743)
Supplement: Supplementary file 3 [file pmen.0000743.s003.docx]

**Response to Reviewers**

**Manuscript ID:** PMEN-D-26-00298

**Title:** Barriers to Substance Use Disorder Treatment among Asian Americans in New York City: A Qualitative Study of Provider Perspectives
**Journal:** PLOS Mental Health

Dear Editors and Reviewer,

Thank you for the opportunity to revise our manuscript and for the constructive and encouraging feedback. We appreciate the reviewer's positive assessment and the editorial guidance, which have strengthened the manuscript. Below we respond to each point in turn. Reviewer and editorial comments are shown in italics, followed by our responses. All changes are reflected in the revised manuscript (marked in the tracked-changes version); page and line references correspond to the tracked-changes file.

# **Journal Requirements**

**1. Ethics statement – documentation and witnessing of verbal consent.**

*In the ethics statement in the Methods, you have specified that verbal consent was obtained. Please provide additional details regarding how this consent was documented and witnessed, and state whether this was approved by the IRB.*

**Response:**

We have expanded the Ethics Statement in the Methods to fully address this point. The revised statement now specifies that the NYU Langone Health IRB (protocol #S22-00857) approved the use of verbal informed consent with a waiver of documentation (written signature) given the minimal-risk nature of the interviews and their remote (Zoom) and in-person conduct. Before each interview, the interviewer (SH or SC) read a standardized, IRB-approved consent script covering the study's purpose, procedures, potential risks and benefits, confidentiality protections, and the voluntary nature of participation, and answered any questions; participants then provided verbal consent both to take part and to be audio-recorded. Consistent with the IRB-approved waiver of documentation, no signed consent form or separate witness attestation was obtained; instead, the interviewer obtained verbal consent directly and, for each participant, noted within the study records that verbal consent had been provided. Written signatures were not collected in order to limit the retention of identifying information.

**2. Financial Disclosure statement.**

*Please amend your detailed Financial Disclosure statement (full sentences; exact wording to be published), clarifying all sources of support with grant numbers and organizations, the initials of the author(s) receiving each grant, the role of the funders, and any salary support.*

**Response:**

We have entered the following statement, in full sentences, into the Financial Disclosure field of the online submission form, matching the funding statement in the manuscript: "This work was supported by the National Institutes of Health and the National Institute on Minority Health and Health Disparities, facilitated by the NYU Center for the Study of Asian American Health (CSAAH; NIH U54MD000538) to SC. The funders had no role in study design, data collection and analysis, decision to publish, or preparation of the manuscript." Per PLOS policy, this statement is entered into the submission system and is not printed in the manuscript body.

**3. Data availability.**

*PLOS requires all data underlying the findings to be freely available, except where public deposition would breach compliance with the approved ethics protocol; if data cannot be shared for ethical/legal reasons, please explain by return email so an exemption request can be escalated to the editor.*

**Response:**

We respectfully request an exemption from public deposition on ethical grounds and have confirmed this by return email to the editorial office. Our data are qualitative interview transcripts from a small number (n = 8) of identifiable providers serving specific Asian American communities in New York City; the transcripts contain contextual detail that could reasonably re-identify participants, and public deposition would breach both the confidentiality assurances given to participants and the conditions of our IRB-approved protocol (NYU Langone Health IRB #S22-00857). Accordingly, the revised Data Availability statement notes that all relevant excerpted data, in the form of de-identified interview quotations, are included within the article, and that de-identified data beyond these excerpts may be made available to qualified researchers for legitimate scientific purposes upon reasonable request to the corresponding author and subject to NYU Langone Health IRB approval (contact: [ogcs.researchcompliance@nyulangone.org](mailto:ogcs.researchcompliance@nyulangone.org)).

**4. Placement of the Ethics Statement.**

*Please ensure that your Ethics Statement is available in its entirety at the beginning of your Methods section, under a subheading ‘Ethics Statement’.*

**Response:**

The Ethics Statement now appears in its entirety at the beginning of the Materials and Methods section, under the subheading "Ethics statement," immediately preceding the Study design subsection.

**5. Supporting Information files.**

*You have Supporting Information legends but no corresponding files uploaded. Please upload them as separate files with the item type ‘Supporting Information’.*

**Response:**

We have uploaded the two Supporting Information files as separate items with the 'Supporting Information' item type: S1 Checklist (COREQ 32-item checklist) and S1 Appendix (semi-structured interview guide). The corresponding legends are retained in the Supporting Information section of the manuscript.

# **Additional Comments**

We thank Reviewer #1 for the careful and encouraging review and for recommending publication following minor revisions. We have addressed each of the three specific comments below.

**Comment 1. Additional literature to support the study rationale (Introduction).**

*The manuscript states that research on SUD and co-occurring mental health needs among Asian American communities remains limited and that few studies have focused on healthcare providers’ perspectives. As these statements provide part of the rationale for the study, they would be strengthened by citing additional relevant literature.*

**Response:**

We have strengthened the rationale in the Introduction with additional supporting citations. The claim that research remains sparse is now supported by our systematic review and related work on the understudied status of SUD among Asian Americans (and the claim regarding the scarcity of provider-focused work is supported by the existing review and barriers literature, including Fong and Tsuang's foundational work on Asian Americans, addiction, and barriers to treatment and Lee et al. We have also incorporated structural-level evidence on the persistent underrepresentation of Asian American, Native Hawaiian, and Pacific Islander participants in NIH-funded research. We would welcome any additional specific studies the reviewer considers essential.

**Comment 2. Clarify whether categories were inductive or predefined (Data analysis).**

*The data analysis section would benefit from clarifying whether the predefined categories were developed inductively during the coding process or were established prior to the analysis, to improve transparency and rigor.*

**Response:**

We have revised the Data analysis subsection to clarify that we used a combined deductive–inductive thematic approach. Broad topic domains corresponding to the four areas of the interview guide (service provision, barriers to care, community needs, and client engagement strategies) were specified a priori and provided an initial organizing framework, whereas the specific codes, themes, and subthemes within and across these domains were not predetermined but were developed inductively from the data during coding. The consolidated matrix therefore organized the inductively derived themes within the a priori topic domains. We have aligned the Abstract wording accordingly.

**Comment 3. Align the thematic structure of Table 2 with the Results section.**

*There appears to be some inconsistency between the thematic framework in Table 2 and the organization of the Results section (e.g., Cultural considerations, Race/ethnicity data collection, and Community-level interventions are incorporated under different headings in the narrative). Aligning the thematic structure across Table 2 and the Results section would improve clarity and coherence.*

**Response:**

We have restructured Table 2 so that it uses the same six themes, in the same order, as the Results narrative: (1) Types of services; (2) Substance use trends; (3) Barriers to care; (4) Community needs; (5) Existing interventions tailored to Asian Americans; and (6) Client engagement strategies. The key points that previously appeared under separate rows (e.g., Cultural considerations; Race/ethnicity data collection; Community-level interventions) have been redistributed without loss of content into these six themes, so that the table and the Results headings now correspond directly.

# **Closing**

We are grateful for the reviewer’s and editor’s time and believe the manuscript is substantially improved. We hope the revised version is now suitable for publication in PLOS Mental Health, and we would be glad to make any further changes the editorial team requires.

Sincerely,

Sugy Choi, PhD, on behalf of all co-authors
