# Supplementary material for: Barriers to substance use disorder treatment among Asian Americans in New York City: A qualitative study of provider perspectives
Source: PLOS Ment Health. 2026 Oct 21;3(10):e0000743. doi: 10.1371/journal.pmen.0000743 (PMC13686486; doi:10.1371/journal.pmen.0000743)
Supplement: Supplementary file 4 [file pmen.0000743.s004.docx]

# Response to Reviewers

Manuscript: *Barriers to Substance Use Disorder Treatment among Asian Americans in New York City: A Qualitative Study of Provider Perspectives*

We thank the editor and reviewers for their careful reading of our manuscript and their constructive feedback. Below we respond to each point raised. Reviewer comments are reproduced in italics, followed by our response. All changes are shown as tracked changes in the accompanying "Revised Manuscript with Track Changes" file and are incorporated into the clean "Manuscript" file.

## Reviewer #1

*Comment: Presentation of findings: The Results section is generally well organized, and the alignment between Table 2 and the narrative has been substantially improved. However, several main themes are introduced primarily through descriptive summaries rather than being immediately supported by representative participant quotations. In particular, the themes Types of services, Barriers to care, and Existing interventions tailored to Asian Americans would benefit from the inclusion of one or two illustrative quotations that directly substantiate the overarching themes before or alongside the presentation of the subthemes. Strengthening these sections with representative participant voices would enhance the credibility, transparency, and richness of the qualitative findings.*

**Response:** We thank the reviewer for this helpful suggestion and agree that representative quotations strengthen the credibility and richness of the qualitative findings. We have added illustrative participant quotations to all three:

- **Types of services:** We added a quotation from a director immediately following the description of the range of services, to directly substantiate the theme before the narrative continues.
- **Barriers to care:** We added an overarching quotation from a counselor at an SUD clinic before the presentation of the cultural stigma, language and communication, and legal/housing/economic precarity subthemes, so that the overarching theme is substantiated up front.
- **Existing interventions tailored to Asian Americans:** We added two quotations: one from a community outreach coordinator illustrating language-concordant staffing, and one from a counselor illustrating the need for stronger legal–treatment collaboration. These are interleaved with the existing narrative on bilingual services, CBOs, and court-mandated programs.

These quotations were drawn from the study's coding matrix and are consistent with the participant roles already cited elsewhere in the manuscript. All changes are marked in the tracked-changes file (Results section, subsections “Types of services,” “Barriers to care,” and “Existing interventions tailored to Asian Americans”).

## Reviewer #2

*Comment: Authors addressed the comments thoroughly. This manuscript now reads smooth and clear. There is one very minor typo that a question mark was left in this sentence: "SUD treatment for Asian American communities in NYC? Reporting follows the Consolidated Criteria for Reporting Qualitative Research".*

**Response:** We thank the reviewer for catching this. We have corrected the typo in the Study design subsection of Materials and Methods. The sentence now reads: “...shape access to SUD treatment for Asian American communities in NYC. Reporting follows the Consolidated Criteria for Reporting Qualitative Research (COREQ) checklist (S1 Checklist).”

We thank the editor and reviewers again for their time and thoughtful comments, which we believe have strengthened the manuscript.
